# A Case of Chondrosarcoma Arising in the Temporomandibular Joint

**DOI:** 10.1155/2015/832532

**Published:** 2015-01-26

**Authors:** Tsutomu Nomura, Tadaharu Kobayashi, Susumu Shingaki, Chikara Saito

**Affiliations:** Division of Reconstructive Surgery for Oral and Maxillofacial Region, Department of Tissue Regeneration and Reconstruction, Course for Oral Life Science, Niigata University Graduate School of Medical and Dental Sciences, Gakkocho-dori 2-5274, Chuo-ku, Niigata 951-8514, Japan

## Abstract

Chondrosarcoma is a malignant tumor originating in cartilaginous cells. And there are only few reports of the case of chondrosarcoma in temporomandibular joint. We discuss a case of chondrosarcoma in temporomandibular joint in a 28-year-old man. Tumor was in contact with the dura, but en bloc resection was performed. After surgical resection of the tumor, face defect was reconstructed by rectus abdominis-free flap. And there is no recurrence after ten years from the resection.

## 1. Introduction

Chondrosarcoma is a malignant tumor originating in cartilaginous cells and retaining this nature throughout its evolution. Next to osteogenic sarcoma, chondrosarcoma is the most common bone tumor [1, 2].

It is rare in the head and neck area especially in the mandible. And there are only few cases of chondrosarcoma in temporomandibular joint. Despite the malignant tumor, chondrosarcoma cases have a long duration from onset to first visit to the hospital [3]. In this case, the duration was ten years and it was one of the longest cases in the collected literatures [4–19].

And treatment of the tumor in the temporomandibular joint is very difficult, because of involvement of facial nerve, parotid gland, and cranial base.

This report describes the details of the case with diagnosis and treatment.

## 2. Case Presentation

A 28-year-old man noticed a swelling of the left preauricular region ten years before presenting at our hospital and simultaneously had a mouth opening limitation.

**Figure 1:**
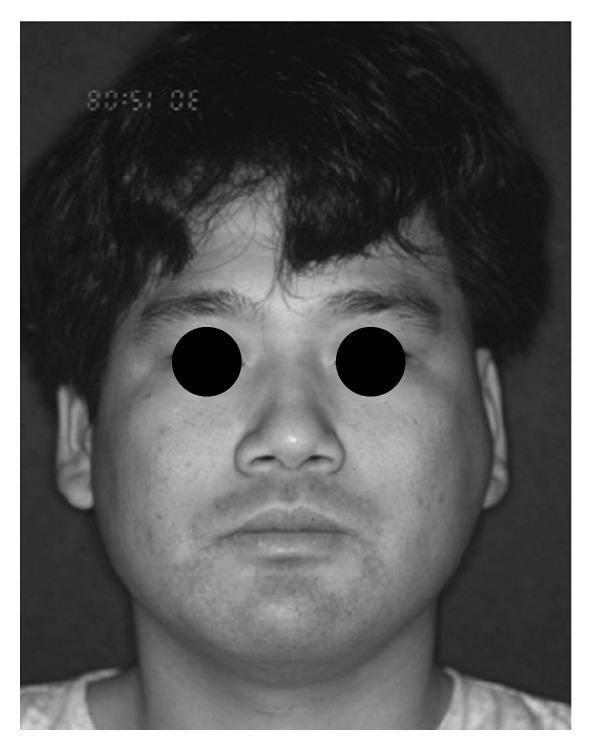
Left side preauricular swelling on presentation.

Although a mouth opening limitation was decreased gradually, he noticed a spontaneous pain of the same region one year before presenting. He visited our clinic, and the patient denied any history of general systemic diseases. The size of preauricular swelling was 40 mm in diameter, but there was no facial paralysis. The lesion was elastic, hard, and tender and was covered with normal skin. There were no abnormal findings about cervical lymph nodes (Figure 1).

The mouth opening was 23 mm between upper and lower incisors, and there was a restriction of both lateral and anterior movements.

**Figure 2:**
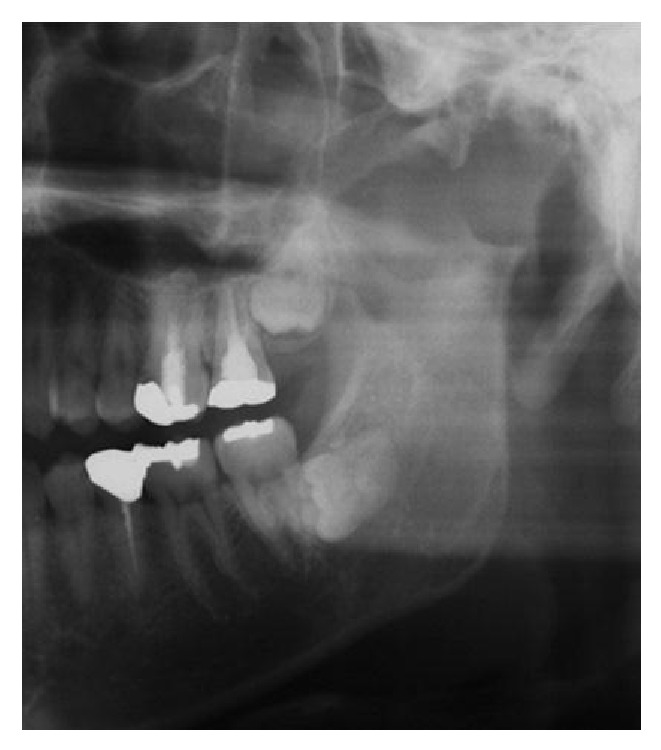
Bone resorption from left fossa to condyle in panorama radiograph.

Panoramic X-ray showed a bone resorption from left fossa to condyle (Figure 2).

**Figure 3:**
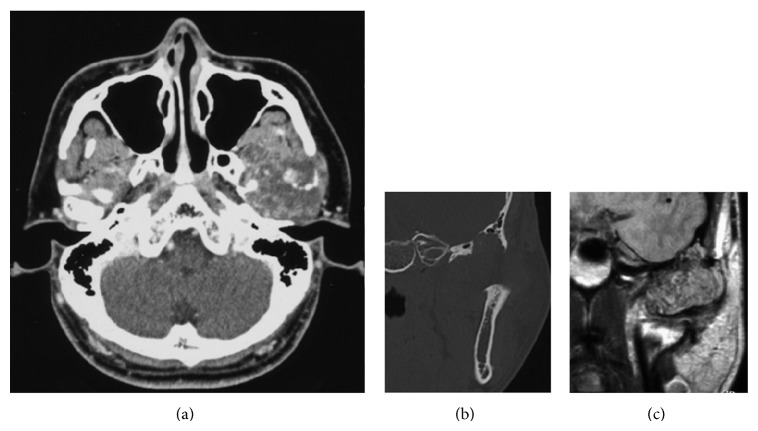
(a) Low-enhanced tumor around the condyle head in contrast-enhanced CT scan; (b) infratemporal fossa erosion and middle cranial invasion of tumor in coronal view of CT scan; (c) tumor expansion close to dura in enhanced MRI.

Computed tomography (CT) scan showed a mass arising from the temporomandibular joint and size was 40 mm in length, 50 mm in width, and 30 mm in height. Enhancement of tumor was low (Figure 3(a)). In superior area, infratemporal fossa was eroded and tumor invaded middle cranial fossa. The mass was seen in cranial area, but invasion of dura was not obvious (Figure 3(b)).

Magnetic resonance imaging (MRI) showed that tumor was expanding close to dura (Figure 3(c)).

To make differential diagnosis, the biopsy was done with preauricular incision. Under the zygomatic arch, the tumor was exposed by collagen capsule incision. The tumor seemed gelatinous. In histological findings, heterogeneous sized tumor cells were embedded in chondrocyte lacuna in the mucinous or chondroid stroma. Although the mitotic figures were not seen, polymorphism of the tumor cells was significant. The diagnosis was chondrosarcoma.

The mass was wide excised and removed from the adjacent structure (temporal process of zygoma, zygomatic process of temporal bone, ramus, and parotid gland) and removed in a single block.

The base of the cranial bone had two bone defects in 5 mm size, but there was no invasion of dura, and the resection of dura was not performed.

In the resected specimen, the disk and lower synovial membrane was seen in intact, and the tumor invaded left parotid gland.

**Figure 4:**
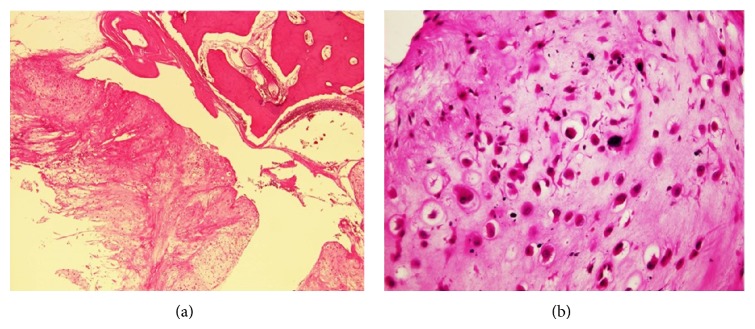
(a) Atypical chondrocytic cells in myxoid or chondroid matrix showing scattered granular calcified materials (×40, HE staining) and (b) pleomorphism with bizarre appearance of nuclei (×400, HE staining).

Microscopic examination showed that the tumor was composed of a diffuse proliferation of atypical chondrocytic cells in myxoid or chondroid matrix where granular calcified materials were scattered (Figure 4(a)). The tumor cells showed cellular pleomorphism with bizarre appearance of nuclei. The mitosis was seen in some area (Figure 4(b)). Histological diagnosis was low grade (Grade 1) chondrosarcoma.

**Figure 5:**
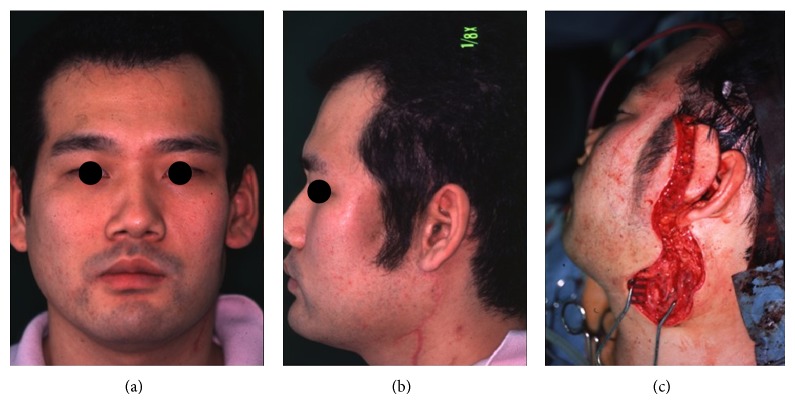
(a) Frontal view of the patient after tumor resection, (b) lateral view of the patient after tumor resection, and (c) intraoperative view of reconstructive surgery.

For the left buccal defect, reconstruction of the left buccal area was done with abdominus cutaneous flap one year after tumor resection (Figure 5). Only soft tissue was reconstructed because of no complaint of mastication.

**Figure 6:**
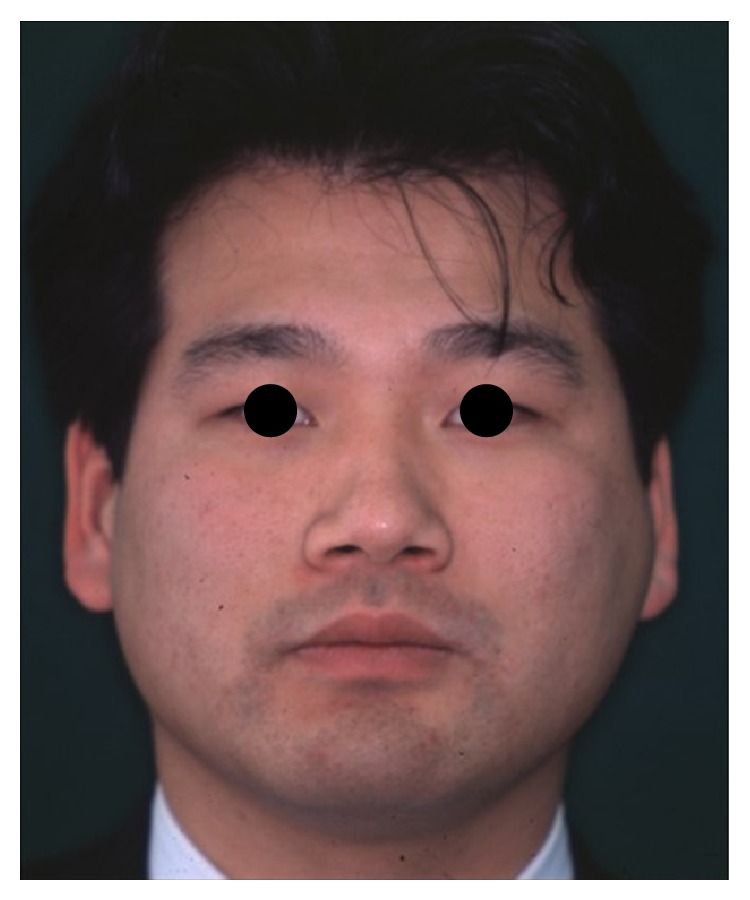
Frontal view of the patient one year after reconstructive surgery.

The recurrence was not seen after ten years from the tumor resection (Figure 6).

## 3. Discussion

Chondrosarcoma is a malignant tumor originating in cartilaginous cells and retaining this nature throughout its evolution. Excluding the report with some missing clinical data, there are only seventeen reports of the cases of chondrosarcoma in temporomandibular joint [4–19] in English literature.

About the age, the mean age of the temporomandibular joint (TMJ) chondrosarcoma is 48 years, and Murayama et al. [20] reported that the mean age of mandible chondrosarcoma is 38 years and Saito et al. [3] reported that it is 41.6 years. The age is higher in TMJ chondrosarcoma group.

And about sex distribution, although Murayama et al. [20] and Saito et al. [3] reported that sex distribution is even in chondrosarcoma in the head and neck area, female is more predominant than male (12 versus 5) in chondrosarcoma in TMJ area.

All these characters of chondrosarcoma in temporomandibular joint are different from chondrosarcoma of head and neck area.

The most major symptom is swelling, followed by pain. Trismus was found in half of the cases. In cases presenting with a preauricular mass, intra-articular tumor can be misdiagnosed as other benign or malignant diseases, such as osteochondromatosis, osteoarthritis, rheumatoid arthritis, avascular necrosis, and condylar fractures. And chondrosarcoma is sometimes misrecognized with temporomandibular disorder. This could be one of the reasons for diagnostic delay.

And some cases had no complaint of pain like our case, and those cases had tendency to have long symptom duration.

And the average of symptom duration of TMJ group is 24 months. It was longer than 8 months in mandible tumor by Murayama et al. [20], 12 months by Saito et al. [3], and 14 months by Weiss Jr. and Bennett [21]. From these data, chondrosarcoma in TMJ seems to have a slow growth character.

To make a correct diagnosis panorama X-ray, CT, and MRI are effective.

Panorama X-ray showed the apparent resorption of condyle head. These findings suggested the tumor in the condyle but not adequate extension area. CT scan showed the invasion of middle cranial fossa and MRI showed tumor expansion close to dura. Performing both CT scan and MRI leads to accurate diagnosis and to making adequate treatment plan.

Cranial invasion was reported by Morris et al. [10], Nitzan et al. [12], and Mostafapour and Futran [15]. We had asked neurosurgeon to back up for cranial surgery. But fortunately we did not need cranial surgery. In case of dura invasion, cooperation with neurosurgeon is imperative.

From Garzino-Demo et al.'s review [17] and our case (Table 1), the symptom duration of the 8 cases that had condyle resorption is 24, 8, 24, 72, 120, 3, 12, and 120 months. These periods were longer than the duration of the cases without condyle resorption.

From this point, destructive potential of chondrosarcoma is almost the same, but the duration could influence the extent of bone resorption.

In terms of diagnosis, fine needle biopsy is not enough for chondrosarcoma from collected literature [15, 18]. And open biopsy is definite to get final diagnosis. Histologically, our case showed Grade I and Nortjé et al. [8], Morris et al. [10], Nitzan et al. [12], Sesenna et al. [13], Garzino-Demo et al. [17], and González-Pérez et al. [18] reported low grade (Grade I) chondrosarcoma. Other reports had no grading information. Our case had a good follow-up course, and all other collected cases have also good prognosis. Murayama et al. [20] reported that 8 of 20 patients died 5 months to 6 years after the primary treatment. Although Evans et al. [22] reported that pathological grade was useful prognostic factor, Saito et al. [3] reported that the grade was not related to the prognosis.

We could not conclude the reason of good prognosis of the chondrosarcoma in TMJ comparing with chondrosarcoma in other head and neck areas, but it might be related to the high rate of Grade I in TMJ chondrosarcoma.

About the treatment, all cases had surgery. As Weiss Jr. and Bennett [21] reported that chemotherapy and radiotherapy are ineffective in chondrosarcoma, wide resection of the tumor is key to the success. Chemotherapy and radiotherapy should be considered in unresected case or positive margin case.

Next to the successful tumor resection, esthetic appearance is very important in head and neck area. Nortjé et al. [8] used sternocleidomastoid muscle (SCM) flap and Nitzan et al. [12] used the temporalis muscle rotation flap for reconstruction.

In our case, because of large defect of cranial base, mandible, and soft tissue, we used rectus abdominis-free flap. This flap has a low donor morbidity. This flap served good esthetic outcome in this case.

## 4. Conclusion

Chondrosarcoma in temporomandibular joint is very rare. Tumor was in contact with the dura, but en bloc resection was performed. Face defect was reconstructed by rectus abdominis-free flap. Open biopsy is definite to get final diagnosis. To make differential diagnosis, panorama X-ray, CT, and MRI are effective.

## Figures and Tables

**Table 1:** Summary of data obtained from the TMJ chondrosarcoma cases reported in the literature and our case.

	Gender	Age	Duration	Symptoms					Appearance at presentation	Treatment	Follow-up
		(years)	(months)	Swelling	Pain	Occlusion	Trismus	Other			(months)
Gingrass (1954) [4]	F	46	12	+	+		+		Slight TMJ space, widening, and subcortical sclerosis	S	—

Lanier et al. (1971) [5]	F	48	24	+	+		+		Condyle resorption	S	—

Richter et al. (1974) [6]	F	75	10	+	+		−	Hearing loss	TMJ space widening, erosion of glenoid fossa, and increased length of condylar neck	S	12

Tullio and D'Errico (1974) [7]	F	17	8	+	−				Condyle resorption	S	—

Nortjé et al. (1976) [8]	M	40	6	+	+		+		TMJ space widening, condyle elongated and distorted	S	24

Cadenat et al. (1979) [9]	F	60	0	+	+				—	S	6

Morris et al. (1987) [10]	F	29	24	+	−		−	Headache	Mass from condyle to infratemporal fossa, cranial fossa eroded	S + RT	6

Wasenko and Rosenbloom (1990) [11]	F	49	0	+	+		−	Hearing loss	Mass from condyle to infratemporal fossa with calcification	S	—

Nitzan et al. (1993) [12]	F	36	72	+	+		+		TMJ space radiolucent lesion, resorption of condyle	S	84

Sesenna et al. (1997) [13]	F	60	12	+	−		+		Mass from condyle to infratemporal fossa with calcification	S	60

Batra et al. (1999) [14]	M	65	18	+	−		−	Hearing loss	Mass anterior to ear canal encasing the mandibular condyle	S	7

Mostafapour and Futran (2000) [15]	F	31	96	+	−				Left pterygoid mass with involvement of TMJ	S	—

Mostafapour and Futran (2000) [15]	F	52	18	+	−				Mass on right TMJ, involvement of petrous temporal bone and middle fossa	S + RT	6

Yun et al. (2008) [19]	F	29	120	−	−	Laterodeviation	+		Mass centered on TMJ with dondylar resorption	S + RT	—

Gallego et al. (2009) [16]	M	54	3	+	+		+		Mass from condyle to infratemporal fossa	S	16

Garzino-Demo et al. (2010) [17]	F	65	3	+	+				Mass centered on TMJ with condylar resorption and calcification	S + RT	9

González-Pérez et al. (2011) [18]	M	57	12	+	+	Open bite, cross bite	−		Erosion of condyle wihtbone destruction	S	24

Our case (2014)	M	28	**120**	+	−		+		Resorption of condyle and cranial fossa eroded	S	120

S: surgery; RT: radiotherapy; TMJ: temporomandibular joint.
